# Study of Mono- and Bimetallic Fe and Mn Oxide-Supported Clinoptilolite for Improved Pb(II) Removal

**DOI:** 10.3390/molecules26144143

**Published:** 2021-07-07

**Authors:** Eva Chmielewská, Wlodzimierz Tylus, Marek Bujdoš

**Affiliations:** 1 Faculty of Natural Sciences, Comenius University, Ilkovičova 6, Mlynská Dolina B2, 842 15 Bratislava, Slovakia; marek.bujdos@uniba.sk; 2 Department of Advanced Material Technologies, Faculty of Chemistry, Wroclaw University of Science and Technology, Wybrzeže Wyspianskiego 27, 50-370 Wroclaw, Poland; wlodzimierz.tylus@pwr.edu.pl

**Keywords:** Fe- and Mn-oxide-supported clinoptilolite, XRD, SEM, XPS, adsorption isotherms

## Abstract

A cost-effective, iron- and manganese-oxide-supported clinoptilolite-based rock was prepared. Based on its nanoporous structure, it worked as a nanoreactor, thereby providing enhanced functionalities. The mono- and bimetallic Fe- and Mn-oxide-supported clinoptilolite was thoroughly characterized with thermoanalytical FT-IR, XRD, SEM, and XPS spectroscopy. All the spectral procedures that were used confirmed the occurrence of a new MnO_2_ phase (predominantly birnessite), including mostly amorphous iron oxi(hydr)oxide (FeO(OH)) species on the surface of the above-synthesized adsorbents. The synthesized products validated a considerably higher adsorption capacity toward Pb(II) pollutants compared to the natural clinoptilolite. The following order of a(max) toward Pb(II) was found: MnOx-zeolite (202.1 mg/g) > FeO(OH)-MnOx-zeolite (101.3 mg/g) > FeO(OH)-zeolite (80 mg/g) > natural zeolite (54.9 mg/g). The adsorption equilibrium data were analyzed by the two-parameter empirical isotherm models Langmuir, Freundlich, and BET as well as the three-parameter Redlich–Peterson isotherm.

## 1. Introduction

Over the few past decades, there has been a dramatic increase in literature data dealing with the design, synthesis, characterization, and property evaluation of zeolites and other advanced materials in a variety of disciplines. In particular, reaction gel chemistry has been considered to play an important role in advanced materials synthesis [1,2,3]. Using mostly the biomimetic sol–gel method for synthesis of iron-oxihydroxide-immobilized zeolite has also been within the scope of numerous scientific papers in recent years [2,4,5]. It is expected that such engineered heterostructures may extend the resultant composite adsorbent with entirely new functionalities or superior efficiency in pollutant removal [4,6]. As is generally known, redox reactions of Fe(II) with Mn(IV) oxides result in an oxidized Fe species, which often occurs as a surface coating on the underlying Mn oxide substrate. It is believed that such metal oxides aggregate in the framework of the host solids and may generate metal clusters, especially on their external surface. Several authors [4,5] have used FT-IR, XRD, SEM, and TEM analyses to determine different Fe oxide precipitates (akaganeite, magnetite, ferrihydrite, or amorphous Fe(OH)_3_ species) onto different Mn oxides (birnessite, poorly crystalline MnO_2_, or the most thermodynamically stable Mn(IV) (pyrolusite)). In fact, the formation of an Fe(III) surface coating on Mn oxide solids would probably impact the rate or overall ability of Mn oxides to remain redox-active. In other words, the Mn oxide surface may become partially passivated. Indeed, Schaefer et al. [6] proved that Fe oxide coatings on the Mn oxide alter its surface properties, but do not shut down the particles’ redox activity.

Although the efficient removal of contaminants has always been problematic in water pollution control, simultaneous oxidation and adsorption onto an FeO(OH)-MnOx-zeolite complex might enable improved performance. Since Mn oxide is utilized in natural water purification for Fe(II) and Mn(II) as well as ammonia removal, biochemical conditions of the process are particularly responsible for acceleration of ammonia concentration drawdown. Therefore, the main role of Mn oxide in water purification is to oxidize Fe (II) species and to remove cations. However, Vinod [7] and Jevtić et al. [8] discovered that alkalic sol–gel treatment of zeolite surface with Fe(III) salts produce mostly Lewis acid sites as well as abundant surface hydroxyl groups that favor adsorption mainly of anionic pollutants in acidic waters. Natural zeolite of clinoptilolite type, which is commonly considered for a cation exchanger, is characterized by a porous structure and negatively charged framework of AlO_4_^5−^- and SiO_4_^4−^-tetrahedra, appropriate for binding or exchanging extra-framework cations. On the basis of previous research, Slovak clinoptilolite [9,10,11] shows a high selectivity toward Pb^2+^. Nevertheless, the chemical modification of its surface allows clinoptilolite to be a more effective adsorbent not only for cationic but also for anionic and even aromatic pollutants [9].

Lead (Pb(II)) pollution in water, coming predominantly from mining, electrotechnical and glass industries, and, especially, battery production, poses a serious threat to human health in many parts of the world. Lead is considered to be one of the most toxic metals with a high bioaccumulation effect through the food chain. Precipitation, ion exchange, coagulation and flocculation, or membrane processes are the commonly applied water treatment and purification techniques for lead removal. However, in the past decades, research has been aimed at developing more efficient and simultaneously cost-effective methods to address this problem. Traditionally, the Fe and Mn oxide-based adsorbents are considered to be effective and low cost adsorption materials for environmental pollutant removal [3,4,5,6]. As they may cause a potential human risk based on nanoscale effect, they need to be immobilized onto various supports (carriers) when their particle size for surface enlargement is below 100 µm [1,2,8]. 

It may be concluded that all the above-mentioned facts contribute to versatile functionalities of recently prepared bimetallic Mn and Fe oxide-supported clinoptilolite-based rock and broaden its potential for pollutant removal. To enhance the understanding of iron and manganese species redox reactions and their interactions with water pollutants, the application of bimetallic-oxide-deposited zeolite with its multiple characterization techniques must be employed. Therefore, the aim of this study was to investigate clinoptilolite surfaces covered with FeO(OH) and MnOx precipitates and their morphology and composition using the accessible analytical techniques (SEM, XPS, XRD, FT-IR, and TG). The aim was furthermore to verify their adsorption properties toward Pb(II), and thus to contribute to the development of new approaches in adsorption-based water purification. 

## 2. Experimental

### 2.1. Adsorbents Examined

Natural zeolite of the clinoptilolite type has been mined since 1970 near Nižný Hrabovec in the neovulcanites region of East Slovakia. The deposit contains rock with a specific mineralogical structure and composition as follows: clinoptilolite 70–85%, volcanic glass 15–20%, feldspar 7–10%, cristobalite 2–4%, and alfa-quartz 2–3% [2,10]. From an economic point of view, this deposit in the Eastern Slovak basin, with an estimated 150 million tonnes of clinoptilolite-based rock is currently the most important reserve in the European Union [11].

The following procedures for MnOx-, FeO(OH)-, and FeO(OH)-MnOx-zeolites synthesis were used: The MnOx-zeolite was synthesized by simple large-scale redox synthesis at ambient temperature and a neutral pH, using a 5% KMnO_4_ and 30% MnSO_4_ solutions. Due to zeolite powdering during the above-described procedures, a narrow grain size fraction of 0.2–0.5 mm was selected for all the experiments using the laboratory sieves (Retsch GmbH, Haan, Germany).

The FeO(OH)-zeolite was prepared as follows: 20 g of (0.2–0.8 mm) grain-sized zeolite was mixed with 0.5 L of 10% aqueous solution of iron(III) nitrate nonahydrate (Fe(NO)_3_·9H_2_O, Alfa Aesar, Crystalline, Germany) and aged at 60 °C in a laboratory water bath shaker for 3 days. Then, 200 mL of 2.5 M KOH solution was added dropwise to prepare the final suspension of pH = 12 and it was aged for about a week at room temperature. After the reaction period the suspension was filtered and washed with deionized water (electrolytic conductivity less than 0.20 µS/cm) and finally dried at 105 °C for 2 h in a laboratory dryer. The FeO(OH)-MnOx–zeolite was prepared as the FeO(OH)-zeolite, only instead of the natural type, the MnOx-zeolite was used as starting material.

### 2.2. Batch Equilibrium Measurements

Batch adsorption experiments were carried out as follows: a 30 mL aliquot of model solution with variable pollutant concentration and 0.3 g of adsorbent (weight with analytical precision) was equilibrated for the designated time interval of 4 h at constant speed (180 rpm) on a Biosan SIA Multi-Rotator in order to reach equilibrium. The supernatant solutions were separated through a 0.45 µm membrane cellulose filter and their residual pollutants analyzed. A sample grain size fraction of 0.2–0.5 mm was used. All measurements were performed in triplicate. The equilibrium uptake capacity a_eq_ (in mg/g) for each sample was calculated according to the following mass balance equation:

(1) $$a_{\mathrm{eq}}=\;\;\left(\frac{C_{i}-C_{\mathrm{eq}}}{m}\right)V$$

where C_i_ and C_eq_ were initial and equilibrium concentrations of the studied pollutant (in mg/L), m was the mass of adsorbent examined (in g), and V was the volume of the solution in liters (L). Chemicals necessary for the Pb(NO_3_)_2_ stock solution preparation were purchased from Lachema Brno (produced in Czech Republic) with analytical grade quality. The pH of stock solutions prepared in DI water ranged from 5.1 to 5.5 depending on the Pb(II) concentration. Lead content of the solutions was determined by flame atomic absorption spectrometry at 283.3 nm with deuterium background correction (AAS Model 1100, Perkin Elmer, Waltham, MA, USA). Calibration standards were prepared from Pb 1000 mg/L stock solution (CertiPUR, Merck, Germany). Measurement range was 0.5–50 mg/L; the solutions with higher Pb concentration were diluted.

### 2.3. Characterization of Analytical Equipment

Thermogravimetric (TG), differential thermogravimetric (DTG), and differential thermal analyses (DTA) were performed on SDT 2960, T.A. instruments by heating 100 mg of the sample at 10 °C/min using the air’s atmosphere in the temperature range 30–1000 °C. All samples were measured in triplicate so that the graphical plots represented their average values. To compare a potential thermoanalytical change between individual samples, their powdered samples were also measured.

X-ray photoelectron spectroscopy (XPS) studies were carried out using a SPECS PHOIBOS 100 hemispherical spectrometer with an Mg source (1253.6 eV) operating at 250 W for high resolution spectra. The spectrometer energy scale was calibrated using the Au 4f_7/2_ and Cu 2p_3/2_ lines at 84.2 and 932.4 eV, respectively. Sample charging was compensated by an electron flood at 0.5 mA current and 0.1 ± 0.01 eV energy. The detection angle regarding the surface was normal. The powdered samples (with a diameter under 100 µm) were pressed into a molybdenum sample-holder. To better understand the nanomorphology of modified zeolites, grain-sized (0.2–0.5 mm) samples, denoted as coarse, were analyzed in the “as received” form or after Ar^+^ sputtering. The C1s peak of the carbon at 284.8 eV was taken as reference in calculating BEs and counting the number of effects. The spectra were collected and processed by SpecsLab II and CasaXPS v. 2.3.19 software. Experimental peaks were decomposed into components (75% Gaussian, 25% Lorenzian) using a nonlinear, least squares fitting algorithm and a Shirley baseline. 

Scanning electron micrographs were taken by Electron Probe Microanalyser JEOL -JXA 840A (Tokyo, Japan) using freshly broken fragments coated with a gold alloy.

FT-IR spectra of FeO(OH)- and MnOx-modified zeolite samples were measured on a FT-IR spectrometer Nicolet 6700 (Thermo Scientific, Waltham, MA, USA). In the IR region of 4000–400 cm^−1^ a DTGS detector and KBr beam divider were used. Measurements were done in transmission mode using pressed KBr tablets (1 mg of sample was homogenized with 200 mg KBr). The spectral analyses were evaluated by means of the program OMNIC.

Mineralogical specification of the adsorbents synthesized was estimated by X-ray diffraction using a Philips Diffractometer (CoKα radiation, voltage 30 kV, intensity 15 mA, Fe filter, diaphragm 1, 1, 05).

## 3. Results and Discussion

### 3.1. Thermal Analysis

**Figure 1:**
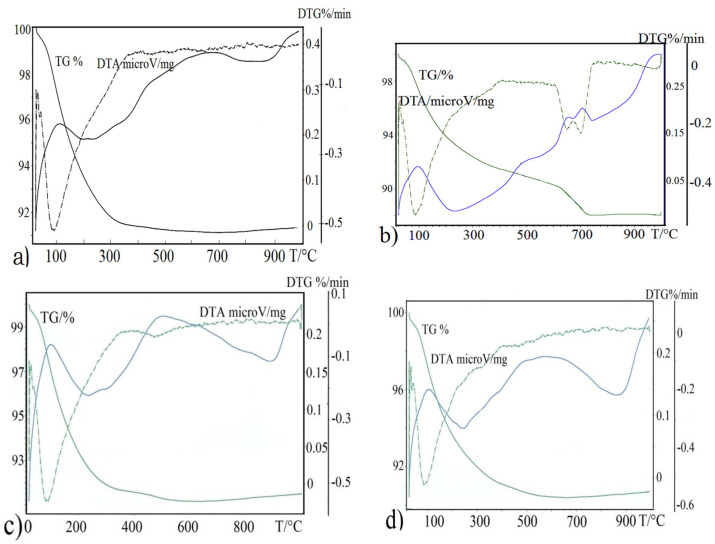
Thermogravimetric analyses of (**a**) natural zeolite, (**b**) MnOx-zeolite, (**c**) FeO(OH)-zeolite, and (**d**) FeO(OH)-MnOx-zeolite.

A powerful thermal (TG, DTA, and DTG) analysis was firstly used for the recently synthesized mono- and bimetallic zeolite modified samples. In general, a decrease in DTA curve can be interpreted as a evaporation, desorption, reduction, and/or decomposition process of the substance examined. According to these thermoanalytical measurements, the sharp endothermic minima under 100 °C were observed in DTA (differential thermal analysis) curves for all zeolite samples (Figure 1a–d). A second endothermic minimum by the FeO(OH)-zeolite appeared at a temperature 500 °C, while no such a minimum was visible by natural zeolite. On the other hand, the DTA curve of MnOx-zeolite (Figure 1b) recorded a strong double split endothermic minimum at a temperature close to 700 °C, which was likely due to the total breakdown of the MnOx-zeolite complex. These mass loss patterns, compared to the other recorded DTA patterns in Figure 1, indicate different thermal behaviors of zeolite modified with manganese oxides. The first shifting of the endothermic minimum to a higher temperature by FeO(OH)-zeolite means a higher water content in the sample after FeO(OH) doping (Figure 1c). Supposing that the Fe(III) metals during the alkalic sol–gel synthesis, replaced some surface Al(III) metals of the skeletal AlO_4_^5−^-tetrahedras or partially destroyed their external framework, the crystal structure of FeO(OH)-zeolite may have started to break down earlier at a temperature below 500 °C. Only the natural zeolite, the FeO(OH)-treated zeolite, and FeO(OH)-MnOx-zeolite recorded increasing endothermic effects in DTG (derivative thermogravimetric) curves around the temperature of 900 °C; for further details, see Figure 1a,c,d). This endothermic effect in the DTG curve shifted to a lower temperature by MnOx-zeolite, just after the exothermic double peak, which was likely characterized by MnOx-zeolite complex destruction. In addition, a potential structural transformation of immobilized manganese oxides (birnessite and pyrolusite), correlated to the resultant extensive weight losses in the DTA curve, should not be excluded. It is supposed that the first endothermic minimum by this modification might be attributed to the loss of water physically adsorbed on the surface and the last one, at the temperature around 700 °C, might be attributed to a drastic structural phase transition (e.g., of the tunnel and layered structures) followed to the total decomposition and collapse of the geocomposite synthesized [2,5,6,12,13].

Simultaneously, based on these DTA patterns, it could be speculated that the intergrowing and interaction of immobilized manganese oxides with zeolitic support, was considerably stronger than by the other modifications. 

### 3.2. FT-IR, XRD, and SEM Studies

**Figure 2:**
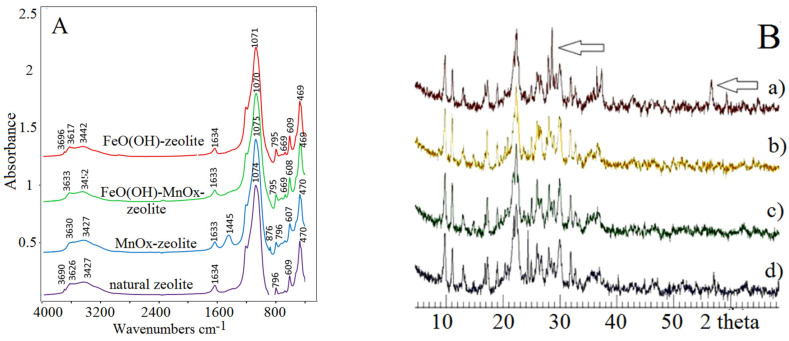
(**A**) FT-IR spectra of samples studied and (**B**) X-ray diffractograms of (**a**) MnOx-zeolite, (**b**) FeO(OH)-MnOx-zeolite, (**c**) FeO(OH)-zeolite, and (**d**) natural zeolite.

**Figure 3:**
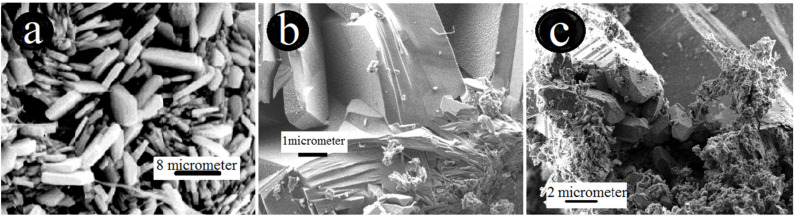
Surface morphology under SEM of natural zeolite-clinoptilolite (**a**), FeO(OH)-MnOx-zeolite, (**b**) and MnOx- zeolite (**c**).

Furthermore, in order to characterize the samples studied in more detail, complementary FT-IR (Fourier-transform infrared spectroscopy), XRD (X-ray diffractometry), and SEM (scanning electron microscopy) investigation of the above geocomposites was performed. As Figure 2A shows, FT-IR spectra of natural zeolite and zeolites enriched with FeO(OH) and MnO_x_ species had characteristic absorption bands in the middle of the IR range, between the wavenumbers of 200 and 300 cm^−1^, observed typically as stretching vibrations of aluminosilicate tetrahedras [8,14,15,16,17,18]. The bands at 1630 and 3690 cm^−1^ were a result of traces of water molecules in the zeolite channels and cavities. Spectra of the newly synthesized FeO(OH)-zeolite also displayed some characteristic peaks at wavenumbers 3696–3617 cm^−1^ and 3442 cm^−1^. Absorption bands at wavenumbers 3442 cm^−1^ by FeO(OH)-zeolite with some reduction and shifting to higher wavenumbers compared to previously investigated pure iron oxihydroxides [2,19], may have indicated physico-chemical surface hydroxylation with O-H.....O groups. However, the FT-IR spectra did not reveal occurrence of any additional crystal phase of Fe oxide in FeO(OH)-zeolite. It is assumed that vibrations at wavenumbers 795 to 796 cm^−1^ by all zeolite modifications, including the natural one, characterize skeletal, crystal-bound Fe(III) oxide content, which is recorded in natural zeolite to ca. 1%. Only the MnOx-zeolite spectrum was enriched characteristically with a 1445 cm^−1^ and a small 876 cm^−1^ absorption band, likely assigning MnO_2_ minerals [20,21,22]. These absorption bands were not ob-served in the FeO(OH)-MnOx-zeolite spectrum, probably due to strong alkalic treatment of MnOx-zeolite and the covering of its surface with FeO(OH). The bands appearing at 400–800 cm^−1^ in the IR spectrum of MnOx-zeolite may have reflected the properties of the Mn-O stretching vibrations [21,22]. The bands in the range of 748–764 cm^−1^, which are missing in our MnOx-zeolite spectrum, are, according to literature [21], the characteristic bands of tunnel-structured pyrolusite. This fact might support the occurrence of birnessite in our sample. The XRD diffractograms did not confirm any clinoptilolite framework destruction nor any structure enrichment with a new Fe crystalline phase (Figure 2(Bb,c)). This recording is typical for well-crystallized clinoptilolite according to the Joint Committee of Powder Diffraction Standards (JCPDS 39-1383). The characteristic Bragg reflections at 10 and 22 and in the range 25–30 of 2 theta indicated the major component clinoptilolite [19]. The deposited FeO(OH) hydrogels in the mesoporous structure of clinoptilolite as the amorphous phase below 10% wt. might be a reason of an indetectable change of both X-ray diffractograms. Only the visible peaks of the primary deposited manganese dioxide (MnO_2_) on the surface of clinoptilolite rock was determined (Figure 3). A potential Bragg reflection around 28 and 57 of 2 theta (Figure 2(Ba)) might identify the crystallized MnO_2_ (e.g., birnessite, pyrolusite, or a potential mixture of manganese oxides) [12,14,22,23].

An inhomogeneous dispersion of Fe(III) and Mn(IV) oxihydroxides in the clinoptilolite rock was deduced. Although the crystal size and detailed morphology of the samples was different, birnessite generally shows an irregular plate-like morphology [21,24]. The morphology of the synthetic K-birnessite sample has a fairly similar SEM image to that of a baerite rose [24]. In this regard, Slovak clinoptilolite contains exchangeable extra framework Ca and K ions in the crystal structure, which may get enriched into predominantly K ions after MnOx-zeolite synthesis [19]. The SEM photomicrograph of FeO(OH)-MnOx-zeolite (Figure 3b) clearly shows typical clinoptilolite morphology, i.e., tabular shaped, about 1 µm sized, crystals with a small external coating right and left at the lower corner of some needle-like (probably akaganeite β-FeOOH) crystals. The irregular and slightly flake-like inclusions or the above-mentioned baerite rose in the Figure 3c in lower part of the image might resemble birnessite δ-MnO_2_ [24], except that there are visible also some typical tabular shaped crystals of the clinoptilolite and probably some larger blocks of manganese oxide prismatic octahedrons.

### 3.3. XPS Investigations

**Figure 4:**
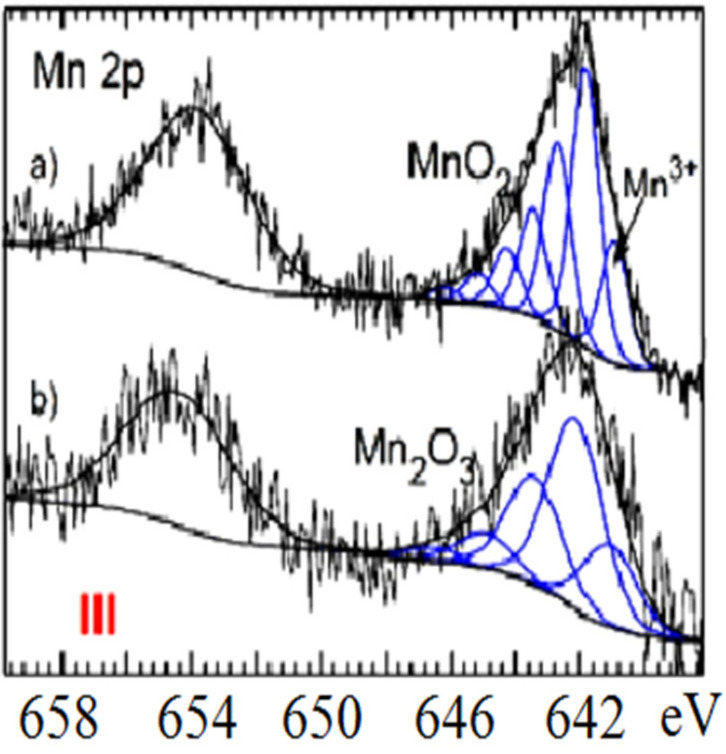
Mn2p core level spectra of (**a**) MnO_2_-zeolite and (**b**) Mn_2_O_3_-zeolite.

XPS analysis helped to describe potentially accessible cations from zeolite channels and cavities as well as the occurrence of various Fe and Mn species with other reaction products on the surface of samples immobilized with manganese oxides (MnOx) and iron oxi(hydr)oxides (FeO(OH)). According to Figure 4 and the observed deconvolution of the Mn2p envelope of MnOx-zeolite, it was concluded that only the MnO_2_ (pyrolusite) compound was present on the surface of such a modified sample. The small peak at the lower BE region of spectrum shows Mn(III) valency. All the measured parameters of the samples investigated are presented in Table 1 and Table 2. Immobilization of FeO(OH) onto MnOx-zeolite caused a significant decrease of Mn content, from 1.04 to 0.37% at. and simultaneously its reduction, probably to Mn_2_O_3_, which corresponds to the parameters presented in Table 1. The Mn2p core level spectra of MnOx-zeolite show at BE 654 eV and of FeO(OH)-MnOx-zeolite at BE 655 eV potential KMnO_4_ peak (Figure 4), probably left from the treatment of samples with KMnO_4_ solution [15,16]. All spectral fitting parameters for Fe 2p3/2, such as binding energy (eV), percentage of the total area (%), and FWHM value (eV) as relative ratios, as well as spectral component separation (eV) originated from the literature cited under [15,16] and are summarized in Table 1 and Table 2.

**Figure 5:**
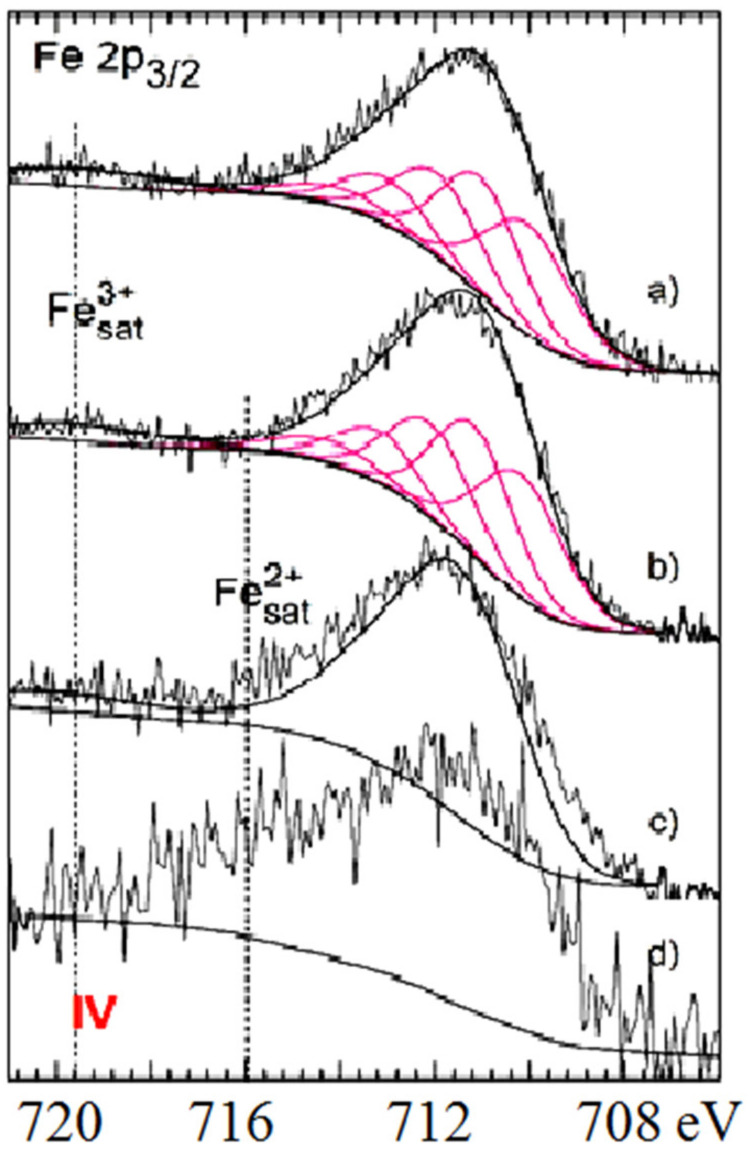
Fe 2p_3/2_ core level spectra of (**a**) FeO(OH)-zeolite, (**b**) FeO(OH)-MnOx-zeolite, (**c**) FeO(OH)-zeolite powdered, and (**d**) natural zeolite.

For both the coarse FeO(OH)-zeolite and FeO(OH)-MnOx-zeolite, as well as according to the deconvolution recorded in Figure 5a,b, an expressive fitting was observed for the present FeO(OH) species. The peak shape of powdered FeO(OH)-MnOx-zeolite (Figure 5c) was different. As observed, the intensity of Fe 2p3/2 photoelectrons was slightly higher in the area of 715–716 eV. Such energy is characteristic for a Fe(II) satellite peak, and probably came from the potential replaced aluminosilicate´s component. Surface oxidation with KMnO4 solution was less invasive on the zeolite structure than the alkalic FeO(OH) treatment, in which the structure of Al(III) ions may undergo some partial replacement with Fe(III) ions. This fact corresponds well with the Si/Al ratio rising from the value of 4.2 for natural zeolite, to 4.5 for MnOx-zeolite, to 5.3 for FeO(OH)-MnOx-zeolite, and to 6.2 for FeO(OH)-zeolite (Table 2). The resulting metal ratios of coarse (0.2–0.5 mm) FeO(OH)-MnOx-zeolite were measured as follows: Mn/Fe = 0.18; Fe/Al = 0.57, and Fe/Si = 0.11. When those results were compared with metal ratios of powdered FeO(OH)-MnOx-zeolite, the ratios of Fe/Al and Fe/Si were lower, i.e., 0.29 and 0.06; however the Mn/Fe ratio was exactly the same value, which means that manganese oxides were not coated with iron oxi(hydr)oxide compounds—otherwise, this Mn/Fe ratio would also have been smaller. It was concluded that Mn(IV) may be localized in deep zeolitic positions or in structure vacancies as chemically bound or as crystalline MnO_2_.

### 3.4. Pb(II) Removal onto Adsorbents Studied

**Figure 6:**
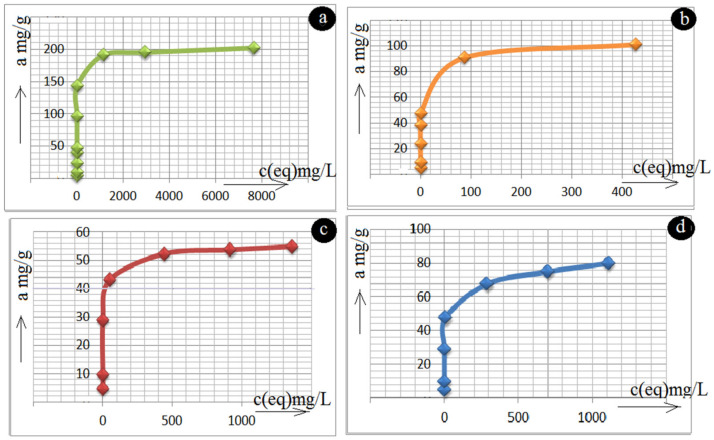
Experimental adsorption isotherms at 23 °C for (**a**) MnOx-zeolite vs. Pb(II) solution, (**b**) FeO(OH)-MnOx-zeolite vs. Pb(II) solution, (**c**) natural zeolite vs. Pb(II) solution, and (**d**) FeO(OH)-zeolite vs. Pb(II) solution.

Table 3 and Figure 6 (isotherms plotted directly from experimental data) present the adsorption results on various surface-modified zeolite samples including the natural one using the statistical regression analysis (Origin version 9.1E, Origin Lab Corp. Linear Fit, Burlington, NC, USA). The equilibrium data from Figure 6 were analysed by two-parameter empirical adsorption isotherm models Langmuir (2), Freundlich (3), and BET (4) as well as with the three-parameter Redlich–Peterson isotherm (5). Results are listed in Table 3:

(2) $$\frac{1}{a}=\left(\frac{1}{a\max.b.c(eq)}\right)+\left(\frac{1}{a\max}\right)$$

(3) $$a=K.c{\left(eq\right)}^{\frac{1}{n}}\;or\;\log a=\log K+\frac{1}{n}\log c(eq)$$

(4) $$\frac{c\left(eq\right)}{a\left(c_{sat}\;-\;c_{eq}\right)}\;=\;\frac{1}{K_{BET}.a_{\max}}\;+\;\frac{K_{BET}-1}{K_{BET}.a_{\max}}\;.\;\frac{c\left(eq\right)}{c\left(sat\right)}$$

(5) $$a\;=\;\frac{A\;.\;c\;\left(eq\right)}{1\;+\;B\;.\;c^{g}\;\left(eq\right)}\;\mathrm{or}\;\ln(A\frac{C_{\mathrm{eq}}}{a}-1)\;=\;g\;\ln(C_{\mathrm{eq}})+\ln(B)$$

where *a* is the specific adsorption capacity in mg/g, *a*(max) is the maximum adsorption capacity in mg/g, *c*(*eq*) is the equilibrium concentration in solution in mg/L, *b* relates to the affinity of the solute for the binding sites expressed in L/mg, *c*(*sat*) is the saturation concentration in solution in mg/L, *K*_(*F*,*L*,*BET*)_ are coefficients of individual isotherms in L/mg, *n* is a heterogeneity factor of the Freundlich isotherm (without unit), and *A*, *B*, and *g* are nonlinear regression constants of the Redlich–Peterson isotherm.

As can be seen from R^2^ (determination coefficient) values, the Langmuir and BET models present much better descriptions of Pb(II) adsorption onto all the samples studied than the Freundlich and Redlich–Peterson isotherm models (Table 3). The Langmuir isotherm describes adsorption on smooth, highly homogenous surfaces, however this isotherm is not applicable for adsorption on surfaces with a lot of irregularities. On the other hand, the Freundlich isotherm contains the heterogeneity factor (n), which relates to the affinity of adsorbing surface, and thus partially compensates for these irregularities. The BET polymolecular adsorption isotherm, unlike to monomolecular Freundlich and Redlich–Peterson isotherms, confirmed a principal advantage in qualitatively adequately fitting the experimental data in a broad concentration range. According to Figure 6 and Table 3 the following order with a maximum adsorption capacity a(max) toward Pb(II) was found: MnOx-zeolite (202.1 mg/g) > FeO(OH)-MnOx-zeolite (101.3 mg/g) > FeO(OH)-zeolite (80 mg/g) > natural zeolite (54.9 mg/g). Based on the results in Table 3, it can be stated that according to calculated determination coefficients (R^2^), the adsorbents examined possess a homogenous surface and adsorption of Pb(II) ions onto their surface fitted better with the polymolecular adsorption model.

The adsorption mechanism of ionic pollutants onto hydrated Fe(III) and Mn(IV) oxides used to be explained by means of the Coulomb, hydrolysis, ion exchange, or coordination sorption models as well as by a combination of all [19,20]. Obviously, in a low pH range of aqueous solutions a positive surface charging predominates on the hydrated oxides. Most authors characterize Fe(III) oxihydroxides as anion-active, i.e., oxides with a positive charging of their surface, while the surface of MnOx is cation-active and therefore has a negative charging [3,5,8,14,20]. This statement is in a good agreement with our results, because a negatively charged MnOx-zeolite showed the highest Pb(II) cation uptake capacity, while the FeO(OH)-zeolite with a positive surface charging showed the lowest adsorption ability for Pb(II) cations. Compared to clinoptilolite alone with its pore size of 0.33 × 0.46 nm, 0.3 × 0.76 nm, and 0.26 × 0.47 nm, the mesoporous nature of the Slovak zeolite rock with plenty of accessible space, much broader external pore openings, and various interparticle voids, provided, during the above synthesis, extensive FeO(OH) and MnOx dispersion, which thus contributed to its increased ability for Pb(II) removal [5,19]. Clinoptilolite alone works as a cation exchanger with an extraordinary high affinity for the Pb(II) cation, i.e., a cation with a rather small cationic radius (1.19 Å) [19,24].

Since the difference in the Pb(II) uptake capacity between natural and FeO(OH)-zeolite was still about 40% (Table 3), the surface of FeO(OH)-zeolite also had to be enriched with sufficient functional groups, which bind Pb(II) cations with higher efficiency than natural zeolite.

With regard to natural zeolite, because MnOx-zeolite reached four-times higher capacity toward Pb(II) ions, coated manganese polyoxides on the external and partially internal zeolite surface, may work as parallel adsorbents. Pb(II) ions from solutions may also intercalate mechanically between its parallel layers (inner sphere complex adsorption), which is typical for some manganese oxide structure (birnessite), and likely also occurs on the zeolite surface (Figure 6, Table 3). According to some literature [21,23,24], synthetic birnessite removes almost an order of magnitude more Pb(II) from solution than the Fe oxides do and the natural pyrolusite removes a similar amount of Pb(II) from solution as many Fe oxides. This statement, correlated with Table 3 and Figure 6, might support the predominant occurrence of birnessite on the surface of our MnOx-zeolite (see also Section 3.2).

Thus, despite of the low pH range of the prepared aqueous solutions (pH 5.1–5.5), when the immobilized Fe oxides on the zeolite were characterized with a positive charging of their surface [20], Pb^2+^ cation removal was also observed. More effective adsorption of FeO(OH)- and FeO(OH)-MnOx-zeolites toward Pb(II) might also be related to the presence of various finely dispersed Fe(III) hydrogels, which bind this pollutant through complexation reactions [17,18,19]. The synthesized iron oxi(hydr)oxide zeolite (FeO(OH)-zeolite) also represents a potential mixture of iron oxides and hydroxides, due to the KOH solution, which was applied during the synthesis to provide high pH and thus allow the iron oxi(hydr)oxide to precipitate.

## 4. Conclusions

(i) Metal oxides such as iron and manganese oxides are natural, mostly fine-grained, low cost adsorbents for aqueous pollutant removal, however their nanoscale counterparts with higher specific surface area must usually be compressed into porous pellets or impregnated onto carriers (such as zeolite) to achieve better filtration and removal performance. From this reason, we prepared and thoroughly characterized the mono- and bimetallic Fe and Mn oxide-supported clinoptilolite in order potentially supply the domestic market with economically feasible adsorbents which can be applied for remediation of old abandoned Pb mining sites in Slovakia.
(ii) The newly synthesized geocomposites exhibited considerably higher adsorption capacity toward the examined Pb(II) pollutant than the natural clinoptilolite. In particular, Mn-oxide-supported zeolite was excellent and was the most efficient adsorbent of Pb(II) from among all the examined samples, showing the highest maximum adsorption capacity toward the tested Pb(II) pollutant of all the samples that have been prepared and variously modified to date in order to improve the adsorption capacity of natural zeolite and thus be offered to the market for water purification.
(iii) According to the thermoanalytical measurements, sharp endothermic minima under 100 °C were observed in DTA curves for all zeolite samples and attributed to the loss of water physically adsorbed. Only MnOx-zeolite recorded a strong double-split endothermic minimum at a temperature close to 700 °C, which was likely due to the total breakdown of the MnOx-zeolite complex. This may have been due to the fact that the intergrowing and interaction of immobilized manganese oxides with zeolitic support was considerably stronger than by the other modifications. Only the MnOx-zeolite FT-IR spectrum was enriched characteristically with a 1445 cm^−1^ and a small 876 cm^−1^ absorption band, likely due to the MnO_2_ mineral (birnessite). After the treatment of raw zeolite with highly alkalic iron nitrate solutions, the XRD measurements did not reveal any clinoptilolite matrix destruction. A potential Bragg reflections around 28 and 57 of 2 theta might identify only the crystallized MnO_2_ (e.g., birnessite, pyrolusite, or a potential mixture of manganese oxides). The irregular and slightly flake-like inclusions or the above-mentioned baerite rose on the scanning electron micrograph in the lower part of the image of MnOx-zeolite might resemble birnessite δ-MnO_2_.
(iv) According to the observed deconvolution of the Mn2p envelope of MnOx-zeolite in the XPS spectrum, it was concluded that only the MnO_2_ (birnessite) compound was present on the surface of such a modified sample. The small peak at the lower BE region of the spectrum also shows Mn(III) valency. Immobilization of FeO(OH) onto MnOx-zeolite caused a significant decrease of Mn content and simultaneously its reduction, probably to Mn_2_O_3_. For both the coarse FeO(OH)-zeolite and FeO(OH)-MnOx-zeolite an expressive fitting was observed for the FeO(OH) species present. It was concluded that Mn(IV) may be localized in deep zeolitic positions or in structure vacancies as chemically bound or as crystalline MnO_2_.
(v) The Langmuir and BET isotherm models presented much better descriptions of Pb(II) adsorption onto all the samples studied than the Freundlich and Redlich–Peterson isotherm models, therefore it can be stated that the adsorbents examined possess a homogenous surface and adsorption of Pb(II) ions onto their surface fits better with the polymolecular adsorption model. The following maximum adsorption capacity a(max) toward Pb(II) was found: MnOx-zeolite (202.1 mg/g) > FeO(OH)-MnOx-zeolite (101.3 mg/g) > FeO(OH)-zeolite (80 mg/g) > natural zeolite (54.9 mg/g). Due to the considerably higher capacity of the MnOx-zeolite toward Pb(II) than that of the FeO(OH)-zeolite, birnessite was simultaneously confirmed as a predominant species in MnOx-zeolite.

## Figures and Tables

**Table 1:** Mn 2p_3/2_ and Fe 2p_3/2_ spectral fitting parameters: binding energy (eV), percentage of total area, and spectral component separation (eV) for given FWHM values (eV).

Compound	Peak 1 BE/FWHM eV	%	Peak 2 BE/FWHM eV	%	Peak 3 BE/FWHM eV	%	Peak 4 BE/FWHM eV	%	Peak 5 BE/FWHM eV	%	Peak 6 BE/FWHM eV	%
MnO_2_	641.8/1.0	41.0	642.7/1.0	27.4	643.5/1.0	16.1	644.3/1.0	8.9	645.2/1.0	4.6	646.2/1.0	2.1
Mn_2_O_3_	640.8/2.0(1.7)	18.9	641.9/2.0(1.7)	44.5	643.1/2.0(1.7)	25.3	644.6/2.0(1.7)	8.5	646.2/2.0(1.7)	3.1		
FeOOH	710.2/2.1(1.4)	27.1	711.2/1.95(1.3)	26.5	712.2/2.1(1.4)	20.5	713.3/2.1(1.4)	11.3	714.4/2.7(1.8)	6.2	719.7/4.3(2.9)	8.3

**Table 2:** Surface composition (% at.) using the XPS method of all the samples studied of grain-size 0.2–0.5 mm.

Sample in % at.	CO_3_^−^	C-O	O 1s	K 2p	Ca 2p	Si 2p	Al 2p	Fe 2p	Mn 2p	Mn/Fe	Mn/Al	Mn/Si	Fe/Al	Fe/Si	Si/Al
Natural zeolite	0.39	0.62	52.5	1.61	1.2	30.04	7.08	0.78	0	0	0	0	0.110	0.026	4.2
MnOx-zeolite	4.14	1.73	49.97	0.64	5.48	19.42	4.29	0.36	1.04	2.89	0.242	0.054	0.084	0.019	4.5
FeO(OH)-MnOx-zeolite	2.83	0.71	51.28	2.42	4.41	19.72	3.71	2.1	0.37	0.18	0.100	0.019	0.566	0.106	5.3
FeO(OH)-zeolite	0	1.34	50.98	2.91	<0.1	28.94	4.65	1.28	0	0	0	0	0.275	0.044	6.2

**Table 3:** Langmuir, Freundlich, BET, and Redlich–Peterson isotherms data for Pb(II) adsorption onto natural, FeO(OH)-, FeO(OH)-MnOx- and MnOx-zeolite.

Adsorbent/Metal Species	Langmuir Isotherm			Freundlich Isotherm			BET Isotherm		Redlich—Peterson Isotherm			
	a(max) mg/g	K_L_ L/mg	R^2^	n	K_F_L/mg	R^2^	K_BET_ L/mg	R^2^	g	A L/g	B (L/mg)^g^	R^2^
MnOx-zeolite/Pb(II)	219.0	1.9114	0.9650	4.5480	37.5742	0.7342	−0.2719	0.9351	0.2189	2.1992	2.4201	0.7575
FeO(OH)-MnOx-zeolite/Pb(II)	102.04	2380.95	0.9995	5.2966	26.9836	0.5669	−2.4015	0.9977	0.2662	6.1494	1.6505	0.7983
FeO(OH)-zeolite/Pb(II)	88.9	0.0083	0.9312	4.4623	19.1381	0.8745	−0.7250	0.9811	0.1805	2.1575	2.0127	0.7424
Natural zeolite/Pb(II)	54.82	0.1289	0.9998	5.1519	15.0.661	0.8083	−0.4068	0.9646	0.2932	1.9867	1.0619	0.6665

## References

[1] Yu L., Liu H., Liu C., Lan H., Qu J. (2016). Magnetically-Confined Fe-Mn Bimetallic Oxide Encapsulation as an Efficient and Recoverable Adsorbent for Arsenic (III) Removal. Part. Part. Syst. Charact..

[2] Chmielewská E., Tylus W., Drábik M., Majzlan J., Kravčak J., Williams C., Čaplovičová M., Čaplovič Ľ. (2017). Structure investigation of nano-FeO(OH) modified clinoptilolite tuff for antimony removal. Microporous Mesoporous Mater..

[3] Simsek E.B., Ozdemir E., Beker U. (2013). Zeolite supported mono- and bimetallic oxides: Promising adsorbents for removal of As(V) in aqueous solutions. Chem. Eng. J..

[4] Jiménez-Cedillo M.J., Olguín M.T., Fall C., Colín A. (2011). Adsorption capacity of iron- or iron-manganese-modified zeolite-rich tuff for As(III) and As(V) water pollutants. Appl. Clay Sci..

[5] Krauklis A., Ozola R., Burlakovs J., Rugele K., Kirillov K., Trubaca-Boginska A., Rubenis K., Stepanova V., Klavins M. (2017). FeOOH and Mn_8_O_10_Cl_3_ modified zeolites for As(V) removal in aqueous medium. J. Chem. Technol. Biotechnol..

[6] Schaefer M.V., Handler R.M., Scherer M.M. (2017). Fe (II) reduction of pyrolusite (ß-MnO_2_) and secondary mineral evolution. Geochem. Trans..

[7] Vinod M., Jinsub L., Jungwon K., Jihyeon G., Alok Kumar R., Jaekook K. (2011). Self-assembled mesoporous manganese oxide with high surface area by ambient temperature synthesis and its enhanced electrochemical properties. Electrochem. Commun..

[8] Jevtić S., Arčon I., Rečnik A., Babić B., Mazaj M., Pavlović J., Matijaševic D., Nikšić M., Rajić N. (2014). The iron (III)-modified natural zeolitic tuff as an adsorbent and carrier for selenium oxyanions. Microporous Mesoporous Mater..

[9] Samuel C.N., Tang I., Lo M.C. (2013). Magnetic nanoparticles: Essential factors for sustainable environmental applications. Water Res..

[10] Barloková D. (2008). Natural zeolites in the water treatment process. Slovak J. Civ. Eng..

[11] (2018). Personal Communication with Project Manager Enviro & Industry A. Cseteová. https://www.zeocem.com/en/products/enviro/zeowater.

[12] Baskan M.B., Pala A. (2011). Removal of arsenic from drinking water using modified natural zeolite. Desalination.

[13] Watkins R., Weiss D., Dubbin W., Peel K., Coles B., Arnold T. (2006). Investigations into the kinetics and thermodynamics of Sb(III) adsorption on goethite (α-FeOOH). J. Colloid Interface Sci..

[14] Doula M.K. (2009). Simultanous removal of Cu, Mn and Zn from drinking water with the use of clinoptilolite and its Fe-modified form. Water Res..

[15] Biesinger M.C., Payne B.P., Grosvenor A.P., Lau L.W.M., Gerson A.R., Smart R.S.C. (2011). Resolving surface chemical states in XPS analysis of first row transition metals, oxides and hydroxides: Cr, Mn, Fe, Co and Ni. Appl. Surf. Sci..

[16] Laszczyńska A., Tylus W., Szczygieł B., Szczygieł I. (2018). Influence of post−deposition heat treatment on the properties of electrodeposited Ni−Mo alloy coatings. Appl. Surf. Sci..

[17] Ruíz-Baltazar A., Esparza R., Gonzales M., Rosas G., Pérez R. (2015). Preparation and Characterization of Natural Zeolite Modified with Iron Nanoparticles. J. Nanomater..

[18] Yuh-Shan H. (2006). Isotherms for the Sorption of Lead onto Peat: Comparison of Linear and Non-Linear Methods. Pol. J. Environ. Stud..

[19] Chmielewská E. (2014). Environmental Zeolites and Aqueous Media: Examples of Practical Solutions.

[20] Pitter P. (2009). Hydrochemie.

[21] Min S., Kim Y. (2020). Physicochemical Characteristics of the Birnessite and Todorokite Synthesized Using Various Methods. Minerals.

[22] Long Y., Ruan L., Lv X., Lv Y., Su J., Wen Y. (2015). TG–FTIR analysis of pyrolusite reduction bymajor biomass components. Chin. J. Chem. Eng..

[23] Kang L., Zhang M., Liu Zo Ooi K. (2007). IR spectra of manganese oxides with either layered or tunnel structures. Spectrochim. Acta Part A.

[24] O’Reilly S.E. (2002). Lead Sorption Efficiencies of Natural and Synthetic Mn and Fe-Oxides.

